# Chronic Hepatosplenomegaly in African School Children: A Common but Neglected Morbidity Associated with Schistosomiasis and Malaria

**DOI:** 10.1371/journal.pntd.0001149

**Published:** 2011-08-30

**Authors:** Shona Wilson, Birgitte J. Vennervald, David W. Dunne

**Affiliations:** 1 Department of Pathology, University of Cambridge, Cambridge, United Kingdom; 2 DBL – Centre for Health Research and Development, Faculty of Life Sciences, University of Copenhagen, Frederiksberg C, Denmark; University of Maryland School of Medicine, United States of America

## Abstract

Chronic hepatosplenomegaly, which is known to have a complex aetiology, is common amongst children who reside in rural areas of sub-Saharan Africa. Two of the more common infectious agents of hepatosplenomegaly amongst these children are malarial infections and schistosomiasis. The historical view of hepatosplenomegaly associated with schistosomiasis is that it is caused by gross periportal fibrosis and resulting portal hypertension. The introduction of ultrasound examinations into epidemiology studies, used in tandem with clinical examination, showed a dissociation within endemic communities between presentation with hepatosplenomegaly and ultrasound periportal fibrosis, while immuno-epidemiological studies indicate that rather than the pro-fibrotic Th2 response that is associated with periportal fibrosis, childhood hepatosplenomegaly without ultrasound-detectable fibrosis is associated with a pro-inflammatory response. Correlative analysis has shown that the pro-inflammatory response is also associated with chronic exposure to malarial infections and there is evidence of exacerbation of hepatosplenomegaly when co-exposure to malaria and schistosomiasis occurs. The common presentation with childhood hepatosplenomegaly in rural communities means that it is an important example of a multi-factorial disease and its association with severe and subtle morbidities underlies the need for well-designed public health strategies for tackling common infectious diseases in tandem rather than in isolation.

## Introduction

In 1960, Walters and McGregor wrote “throughout the tropical belt hepatic disease is rife; its aetiology has been attributed severally to malaria, to malnutrition, to infection or to the combined action of all three” [1]. Half a century later it could be argued that in the tropics hepatic disease is still rife and, although widespread, our understanding of the aetiology, underlying mechanisms, and consequences remains poor. Schistosomiasis is a common cause of hepatosplenomegaly in the tropics, with its severest morbidity being hepatosplenomegaly associated with gross periportal fibrosis, also known, after the clinician who first described it, and its distinctive appearance, as Symmer's pipe-stem fibrosis. However, the introduction of portable ultrasound showed that for the majority of schoolchildren in schistosomiasis-endemic areas, the mechanism underlying hepatosplenomegaly is not periportal fibrosis. Here we assess recent clinical and immunological evidence, along with that published in the 1960s, ‘70s, and ‘80s, for dissociation of periportal fibrosis from presentation with hepatosplenomegaly in school-aged children—a morbidity that is now encompassed by the World Health Organization (WHO) Working Group on Schistosomiasis' definition of “subtle” morbidity [2]. We hope to highlight that schistosomiasis mansoni–associated morbidity is not predominantly periportal fibrosis and that another, widespread morbidity, influenced not only by schistosomiasis but other infectious agents, is affecting the lives of millions of children in sub-Saharan Africa.

## Methods

Data for this review was collected by searches in NCBI PubMed and from the references of relevant articles. Numerous articles were identified from the extensive files of the authors. The population-based studies included examine either a broad age range from school-aged through adulthood, or concentrate solely on morbidity of school-aged children. The criteria were relaxed for references relating to immune responses associated with periportal fibrosis when studies examining adult cohorts are referenced.

## Schistosomiasis-Associated Periportal Fibrosis

As the definitive site of *Schistosoma mansoni* is the mesenteric veins, it is necessary for eggs to transverse the gut wall, causing gut pathology associated with diarrhoea, sometimes bloody, and abdominal pain [3]. However, released eggs can be swept by the host's circulation into the liver, becoming trapped. The immune response to trapped eggs, characterised by T helper 2 (Th2)-mediated granulomatous reactions can, over years, cause periportal fibrosis [4]. Long-term deposition of thick fibrotic material around the hepatic vasculature restricts blood flow, leading to portal hypertension. This is accompanied in early stages by a firm, enlarged liver with a smooth surface, while in later stages the liver can become smaller and nodular. The symptoms of portal hypertension—passive congestion of blood flow leading to splenomegaly, presence of ascites and development of varices in collateral veins, and, when these rupture, haematemesis—have all been observed in severe hepatosplenic schistosomiasis.

In a series of autopsy studies carried out by Alan Cheever in the 1960s and 1970s on *S. mansoni*–associated hepatic pathology, in all deaths caused by complications of portal hypertension, gross periportal fibrosis was present [5], [6], with haemorrhaging of oesophageal varices being the most common cause of death [5]. Periportal fibrosis was linked to infection intensity, as indicated by worm recovery on blood perfusion [6], [7]. Infection intensity is not the sole factor in development of severe hepatic schistosomiasis. The autopsy studies reported differences in prevalence of periportal fibrosis between ethnic groups [5], suggesting that development of periportal fibrosis is under genetic control. It is now known that North Africans are more susceptible to periportal fibrosis than sub-Saharan Africans, even when infection intensities are greater in sub-Saharan cohorts [8], [9]. Familial patterns of disease also occur [10] and genetic studies have identified gene polymorphisms associated with periportal fibrosis [11], [12].

## Schistosomiasis–Associated Morbidity in Endemic Communities

Cheever said of his findings, “trends existing in the community of an endemic area may not be evident in this autopsy series, and vice versa.” Prior to the introduction of ultrasound, severity of *S. mansoni*–associated morbidity was accessed using the presence and extent of hepatosplenomegaly by palpation. After the findings of Cheever's work, enlargement of the liver was thought to be due to periportal fibrosis and enlargement of the spleen due to passive congestion and reticuloendothelial hyperplasia [4]. In several community epidemiology studies conducted in different parts of the world, and among different ethnic groups, prevalence of hepatomegaly peaked in older children and adolescents, the age group in which *S. mansoni* infection intensity peaks [10], [13]–[15]. The prevalence of hepatomegaly also increased as *S. mansoni* infection intensities increased [10], [13]–[17]. Clinical presentation was of dominant left liver lobe enlargement, of firm consistency [18], with a significant relationship between extent of enlargement and infection intensities being reported [13], [14], [16].

**Figure 1:**
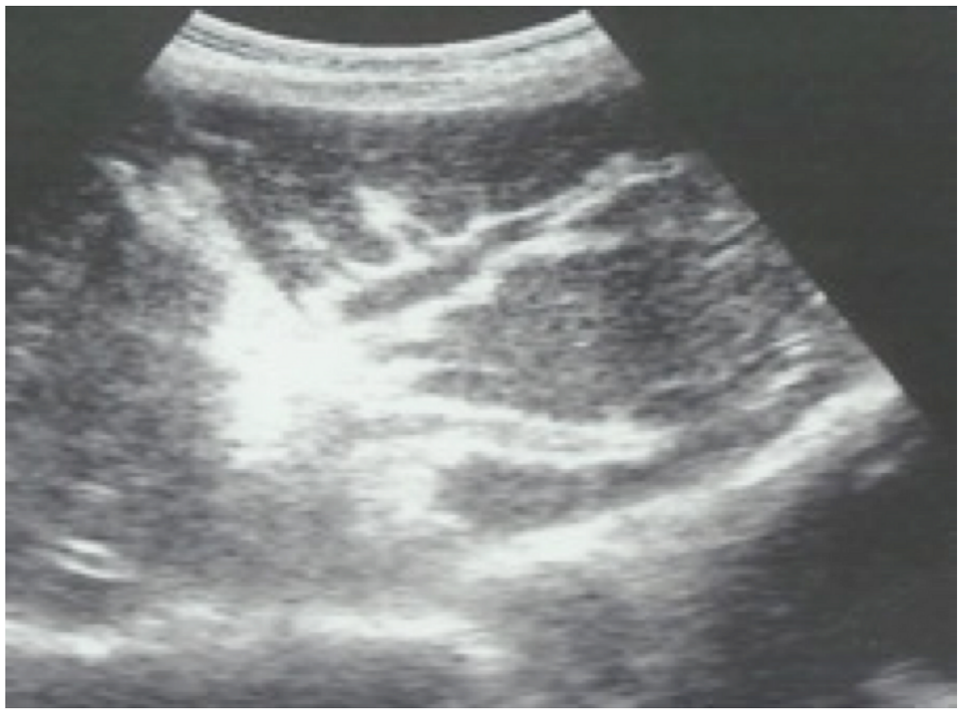
Ultrasound image of periportal fibrosis in *S. mansoni* infection. The image was taken during ultrasound examination of a study cohort from Piida village on the shores of Lake Albert, Uganda, in March 2004 and illustrates liver texture pattern C with “pipe-stems.”

In the 1980s portable ultrasound machines were introduced into epidemiological research of schistosome-associated morbidity. Ultrasonography is now the method of choice for detecting periportal fibrosis in epidemiological studies, as it is distinctive and clearly visible (Figure 1), and sensitivity is such that mild cases can be detected [19]. An international protocol has been established and extensively tested in various endemic situations [20]. The protocol is based on coding of imaged patterns; grades C and D indicate mild fibrosis that often resolves with treatment, and grades E and F represent gross fibrosis, often with sequealae related to portal hypertension, that rarely resolves with treatment [21]. Under the protocol a series of other measurements—liver and spleen size, wall thickness of the second order portal branches, and portal vein diameters—are taken. Presence of collaterals and ascites are also recorded.

**Figure 2:**
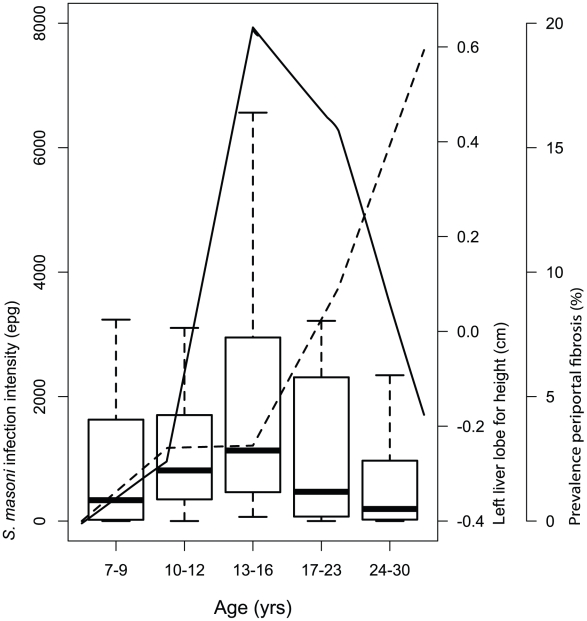
Comparison of *S. mansoni* egg counts (boxplots), liver size (solid line), and prevalence of severe, non-reversible (pattern E/F) periportal fibrosis (dashed line) by age. Data is from a randomised cohort selected from the inhabitants of Booma village on the shores of Lake Albert, Uganda. The sole criterion for selection was born in Booma or over 10 years of residence. The left liver lobe was measured in the parasternal line by ultrasound and was standardised for height by linear regression. A comparison of morbidity amongst a larger population from this village and the neighbouring one is published in detail in [24].

Utilisation of ultrasonography in community-based studies showed that prevalence of periportal fibrosis peaks in adulthood, with cases detectable into middle age, even when *S. mansoni* infection intensity drops dramatically in early adulthood (Figure 2) [22]–[25]. Some studies do, in agreement with the autopsy studies, find an association between periportal fibrosis and *S. mansoni* infection intensities; however, the strength of *S. mansoni* intensity's influence on the presence of periportal fibrosis is weak in comparison to that of age and sex [9], [23], [25], [26], and other studies fail to find an association [8], [22], [27], [28]. The lack of consensus about the influence of infection intensities on periportal fibrosis and periportal fibrosis occurring in adulthood, often in the fourth and fifth decades of life [9], [22], [23], indicate that development of fibrosis is largely dependent on long-term exposure to *S. mansoni*, a conclusion supported by duration of residence in endemic areas, rather than age per se, being strongly associated with presence of periportal fibrosis [24]. Due to the importance of duration of infection in the development of periportal fibrosis, it is rare in school-aged children and when present is usually mild [9], [23], [24], [26], [29].

Taking an overview of clinical-based studies carried out in the 1970s and ‘80s and ultrasound studies conducted from the late 1980s onwards allows us to see that within communities the prevalence and extent of hepatosplenomegaly with age, in the majority of endemic situations, mirrors the characteristic infection intensity curve, while periportal fibrosis is more common in an older age group (Figure 2). Hepatosplenomegaly is often significantly associated with *S. mansoni* infection intensities, a relationship that has been harder to establish for periportal fibrosis. Studies that have used clinical and ultrasound examinations in tandem confirm that many children with *S. mansoni*–associated hepatosplenomegaly show no signs of periportal fibrosis [28], [30], [31]. This supports the proposal made in 1987 that “juvenile hepatomegaly is probably not due to a definitive fibrotic process, but rather to immune responses” [15].

## Childhood Hepatosplenomegaly: An Inflammatory Process

The mechanism behind periportal fibrosis is likely related to granulomas that form around eggs trapped within the liver, a process well researched in the mouse model. In the mouse IL-13 is important, upregulating arginase-1 in granuloma macrophages. Arginase-1 uses arginine to make L-ornithine, an intermediate of an essential amino acid in collagen production, proline, while IFNγ upregulates the production of inducible nitric oxide synthase, a pathway that uses arginine as a substrate, inhibiting the production of proline [32]. In humans, little IL-13 is produced by whole blood cultures stimulated with schistosome egg antigen (SEA), suggesting that IL-13 production is successfully down-regulated in the majority of individuals [33], [34]. However, higher levels of SEA-specific IL-13 [35], [36] and lower levels of SEA-specific IFNγ are associated with periportal fibrosis [37], [38]. Periportal fibrosis is therefore likely due to a sustained Th2 response [39]. In contrast, children who present with hepatosplenomegaly in the absence of periportal fibrosis have low levels of SEA-specific Th2 cytokines, IL-4, IL-5, and IL-13 [40]. Instead, childhood hepatosplenomegaly is associated with high levels of TNF and IFNγ in peripheral blood mononuclear cell cultures [41], and SEA-stimulated whole blood cultures predominantly produced TNFα and the regulatory cytokines IL-6 and TGFβ. Lower levels of IL-6 and TGFβ to schistosome antigen preparations are associated with substantial enlargement of the liver, suggesting a potential lack of regulation [40].

## The Role of Co-Exposure to Malaria

Hepatosplenomegaly can occur during acute malaria attacks, due to reticuloendothelial and lymphoid hyperplasia [42]. Around half of cases of hepatosplenomegaly associated with acute malaria resolve within 2 weeks of treatment and others have partial regression within the same time scale [43], [44]. When treatment fails to clear parasitaemia, or when time between attacks is too short for full resolution, hepatosplenomegaly is persistent [43]. As immunity to malaria develops, time between malaria attacks increases and a corresponding drop in prevalence of hepatosplenomegaly occurs [42], [45]. In community studies in The Gambia during the 1950s and 1960s, age-prevalence curves of hepatomegaly followed the pattern of the age-prevalence curve for malaria parasitaemia. The consistency of the liver was of three types: soft, common in infants; rubbery with a defined edge, most prevalent in children 1–6 years of age; and firm to hard, often of considerable enlargement and most prevalent in older children, adolescents, and adults [46]. Needle biopsy material from children with firm to hard hepatomegaly showed coarse fibrosis around periportal tracts, but no excess in cellular infiltration or deposits of malaria pigment [47]. Young children also had localised fibrotic out growths but, in contrast to older children, they also had lymphocyte infiltrations in hepatic sinusoids, hyperplasia of Kupffer cells, and deposits of malaria pigment were present [1].

With ongoing chronic exposure to malaria being an aetiological agent of hepatosplenomegaly in school-aged children, the age group in which the prevalence and extent of schistosomiasis-related hepatosplenomegaly is also at its peak, the two infections could exacerbate the hepatosplenomegaly caused by each other. In one early study, infection with both parasites, rather than either infection alone, was associated with the presence of hepatosplenomegaly [48]. Latterly, this has been addressed further in a series of studies on childhood hepatosplenomegaly conducted in the neighbouring districts of Machakos and Makueni in Kenya.

**Figure 3:**
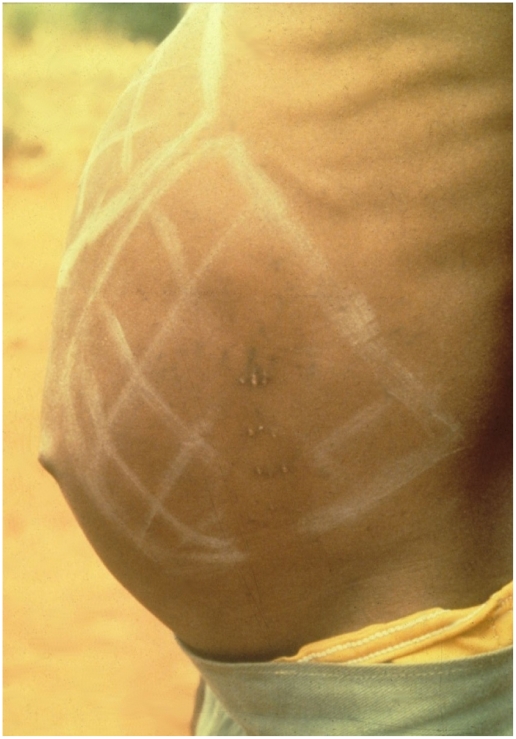
Hepatosplenomegaly in a school-aged girl with schistosomiasis, Kambu, Makueni District, Kenya. The photograph was taken during field studies carried out in 1988. Extent of palpable organs are indicated by chalk markings. The scarification seen is from “treatment” of organomegaly administered by traditional healers. The traditional healer will make small cuts at the edge of the palpable organ. When several rows of scars are seen (as shown), this represents repeat “treatment” and therefore shows the progression of organ enlargement with time.

The first, a cross-sectional study, compared prevalence of hepatosplenomegaly in school-aged children from Kangundo, Machakos District, and Kambu, Makueni District. *S. mansoni* infection intensities were comparable, and prevalence of hepatosplenomegaly within both communities was significantly associated with *S. mansoni* infection intensities, but prevalence was greater in Kambu [49]. In Kambu some cases of hepatosplenomegaly were very severe (Figure 3), and the children were hospitalised for further examination. Interactions between *S. mansoni* and common gut parasites could not explain the difference as there was no significant difference in prevalence of the common gut helminths or protozoa, and there was no association between the presence of *S. mansoni* and any of the gut parasites [50]. One major difference between Kangundo and Kambu, due to altitude, is the importance of malaria as a public health problem. Although current microscopically detectable malaria parasitemia was not directly associated with hepatosplenomegaly, it was suggested that chronic exposure to malaria could be exacerbating hepatosplenomegaly [49]. This suggestion was substantiated by a case control study in which children with hepatosplenomegaly had elevated plasma levels of IgG3 specific for *Plasmodium falciparum* schizont antigen (Pfs) [51]. Pfs-IgG3 levels are representative of relative age and geographical exposure to malarial infections [52].

In children selected on the basis of having an enlarged liver, both the extent of the liver and the spleen were significantly associated with *S. mansoni* infection intensities. Ultrasound examinations showed that hepatosplenomegaly was present in the absence of periportal fibrosis [30]. During a 3-year follow-up period, although significant regression of hepatosplenomegaly occurred post-treatment for schistosomiasis, full resolution of hepatosplenomegaly did not occur in all children, even though the Kambu River, the one site of *S. mansoni* transmission, had been mollluscicided regularly, limiting population numbers of the *Biomphalaria* snail intermediate host [53]. Pfs-IgG3 responses were elevated in children who lived close to the Kambu River and it was these children, who also had high *S. mansoni* infection intensities, who had the largest spleens at baseline [54] and the poorest rate of resolution [55]. Finally, children in two neighbouring primary schools, one with a catchment area with *S. mansoni* transmission and the other in an area where *S. mansoni* transmission is abrogated due to the lack of habitat for the intermediate host, presented with a comparable prevalence of hepatosplenomegaly. The presence of hepatosplenomegaly was associated with plasma levels of Pfs-IgG3, but children with *S. mansoni* infections had greater liver and spleen enlargement; for the liver this exacerbation was *S. mansoni* infection intensity dependent [31].

## Exacerbation of the Inflammatory Process with Co-Exposure to Schistosomiasis and Malaria

Higher levels of the pro-inflammatory cytokine IL-12p70 in plasma are associated with hepatosplenomegaly [40]. Levels of the regulatory cytokine IL-10 and soluble TNF receptor I (sTNF-RI) and sTNF-RII are also significantly higher in children with hepatosplenomegaly [40], [41]. As sTNF-RI and sTNF-RII are antagonistic soluble receptors, induced by TNFα itself [56], [57], and IL-10 is induced during a pro-inflammatory response as a feedback control mechanism [58], [59], it is likely that these responses reflect an attempt to control a pro-inflammatory response. In Mali, sTNF-RII levels are elevated in children co-infected with *P. falciparum* and *Schistosoma haematobium*, either in comparison with children who only have malaria [60], or in comparison with children who only have *S. haematobium* [61]. IL-12p70, IL-10, and sTNF-RII are strongly correlated with the Pfs-IgG3 malaria exposure marker, and levels of IL-10 and sTNF-RII are significantly greater in children who are co-infected with *P. falciparum* and *S. mansoni* [62]. Furthermore, Th2 responses to SEA are down-regulated by *P. falciparum* infection [40]. The results of cytokine analysis, from cells stimulated with schistosome antigens *ex vivo* and from plasma levels, therefore, indicate that childhood hepatosplenomegaly is associated with a pro-inflammatory response to which both *S. mansoni* and *P. falciparum* infections contribute. Whether the exacerbation is purely additive or synergistic, remains to be clarified; however, the down-regulation of SEA-specific Th2 responses in children infected with *P. falciparum*, a finding also reported from mouse models of malaria/schistosome co-infection [63], , does indicate interaction at the immunological level between the two parasites.

## Sequelae of Childhood Hepatosplenomegaly

During an early cross-sectional study in Maukeni District, some cases of hepatosplenomegaly in Kambu were of a severity that the children were hospitalised for further examination. Eighteen were found to have oesophageal varices, a sub-set of whom did not have ultrasound detectable periportal fibrosis [49]. Systematic ultrasound assessment in later field studies showed that none of the children included had detectable periportal fibrosis [30], [31]. However, following fully the Niamey protocol to analyse data, with comparisons being made to *S. mansoni* uninfected Senegalese who serve as a standard reference population, 28% of the children examined were classed as having portal hypertension. In a later cross-sectional study, due to ongoing debate as to the suitability of the Senegalese reference population and the reproducibility of some of the more difficult measurements [9], [65], [66], inter- and intra-observer robust portal vein diameter measurements [65], standardised internally for height, were used and were both greatest amongst children who had hepatosplenomegaly, and related to Pfs-IgG3 levels and *S. mansoni* infection intensities [67]. The long-term consequences of this dilation of the portal vein are poorly understood, but previous findings that ascites and varices can be present in the absence of periportal fibrosis indicates that “subtle” morbidity, in this case hepatosplenomegaly, may have health consequences that are far from subtle.

As long-term insults to health can result in stunting of growth [68], [69], and childhood hepatosplenomegaly is of a chronic nature, nutritional status has been assessed. Comparison of the nutritional status of children in a high morbidity area with that of children in the low morbidity area showed poorer height-for-age amongst the former, being worst in children with hepatomegaly. However, diet was also poorer and as it could not be determined whether poor nutrition was involved in the development of hepatomegaly, or hepatomegaly was causing poor nutritional status, nutrition could not be discounted as an aetiological agent of hepatomegaly [70]. More recently poor height-for-age, determined by comparison with the CDC 2000 standards, was found to be highly prevalent, with 59.4% of children in an endemic area being classified as stunted. Stunting was most prevalent amongst children with organomegaly, reaching 75.4% amongst children positive for *S. mansoni* and with hepatosplenomegaly. In the interim between these two studies a school-feeding program was introduced, removing the influence of current poor nutritional intake [67]. The implication is that the underlying process causing hepatosplenomegaly is also having a detrimental effect on the growth of children. The mechanism is purely speculative, but one proposal is that inflammatory cytokines produced during childhood hepatosplenomegaly are adversely effecting the production of the growth hormone insulin growth factor one by the liver.

## Other Potential Aetiological Agents

In the studies carried out in Machakos and Makueni Districts, visceral leishmaniasis was discounted as no outbreaks occurred during the 15 years that this series of studies spanned; and although investigated by researchers at the Kenya Research Institute, there is no evidence that brucellosis is contributing, even though contact with livestock is the norm (A. E. Butterworth, personal communication). Other infectious agents include the chronic hepatitis viruses B and C. In the Machakos/Makueni studies no cases of discompensated cirrhosis were observed, and fine, compensated cirrhosis is not detectable by ultrasound. However, in many of the African populations studied the prevalence of hepatosplenomegaly is too great, and the age group in which it is observed too young, for viral hepatitis to be a major contributing factor [71], [72].

Although circumstantial evidence, there was an observed improvement in the health of children from Makueni after the introduction of the school-feeding program subtle, long-term health consequences were observable, but the severe consequences of ascites and varices were no longer seen. Poor nutrition therefore cannot be discounted as an important modifier of hepatosplenomegaly and its consequences.

In 1987, Kevin De Cock, working at the Kenyatta National Hospital in Nairobi, reported that Akamba, the main tribal group in Machakos and Makueni Districts, had a higher incidence of idiopathic portal hypertension than other tribal groups in Kenya. He concluded that this could be attributable to an environmental cause [73]. This tribal group is also reported to have a higher incidence of hepatocellular cancer [74]. Chronic aflatoxin exposure is known to increase the risk of hepatocellular cancer [75], though it is not known whether chronic aflatoxin exposure causes hepatomegaly in the absence of hepatocellular cancer. In Machakos and Makueni Districts, as in large parts of sub-Saharan Africa, maize is the staple food source, and this crop, along with groundnut, another common food staple, is particularly susceptible to growth of *Aspergillus spp.*, the fungus that produces aflatoxin [75]. We have recently undertaken collaborative analysis to determine the role of chronic exposure to aflatoxin, and results indicate that aflatoxin is contributing to the hepatosplenomegaly observed (Y-Y Gong, unpublished data). School-aged hepatosplenomegaly is therefore an example of a multi-factorial disease, attributable to a number of aetiological agents that are associated with poverty and widespread in sub-Saharan Africa.

## Conclusions

By utilising new tools such as portable ultrasound machines, identification of serological markers, and good international standards on child growth, our understanding of the causes and consequences of childhood hepatosplenomegaly in the tropics has improved. It is clear that hepatosplenomegaly associated with schistosomiasis mansoni, in the absence of periportal fibrosis, is a distinct widespread morbidity amongst school-aged children that can have severe health consequences. Advances in immunological reagents and instrumentation allow us to measure many more mediators in small quantities of plasma or cell culture supernatants, giving insight into potential dysregulation of a pro-inflammatory process. The continuation of good epidemiological study design techniques, in combination with new satellite GIS techniques, has highlighted the importance of confounders or modifiers. A particularly important finding is the role of chronic exposure to malaria in exacerbation of *S. mansoni*–associated hepatosplenomegaly, through the potential modulation of the inflammatory process. Exacerbation by chronic exposure to malaria underlines the importance of assessing as many potential confounders as is logistically feasible. As Walter and McGregor noted in 1960, the aetiology of childhood hepatosplenomegaly is complex, but we now have the computational and statistical tools to take a more holistic approach. To study an infection in isolation is an opportunity missed to broaden our knowledge of this so-called subtle morbidity, its underlying causes, mechanisms, and long-term health consequences. Equipped with this knowledge, rather than burdening children with the residual consequences of treating causative agents in isolation, informed integrated public health policies could be designed with the aim of maximising alleviation from this widespread and debilitating morbidity.

**Key Learning Points**

- Age-prevalence profiles of *S. mansoni*–associated hepatosplenomegaly peak in adolescence, corresponding with the peak in *S. mansoni* infection intensity, whilst periportal fibrosis age-prevalence profiles peak in an older age group.
- *S. mansoni*–associated childhood hepatosplenomegaly has been observed in the absence of periportal fibrosis.
- Periportal fibrosis is associated with high levels of specific Th2 responses, whilst childhood hepatosplenomegaly is associated with high levels of Th1 responses.
- *S. mansoni*–associated childhood hepatosplenomegaly is exacerbated by concurrent exposure to *Plasmodium* infections.
- Childhood hepatosplenomegaly is not benign, with both dilation of the portal system and stunting of growth being associated.

**Key Publications**

- Cheever AW (1968) A quantitative post-mortem study of Schistosomiasis mansoni in man. Am J Trop Med Hyg 17: 38–64.
- Lehman JS Jr, Mott KE, Morrow RH Jr, Muniz TM, Boyer MH (1976) The intensity and effects of infection with *Schistosoma mansoni* in a rural community in northeast Brazil. Am J Trop Med Hyg 25: 285–294.
- Homeida M, Ahmed S, Dafalla A, Suliman S, Eltom I, et al. (1988) Morbidity associated with *Schistosoma mansoni* infection as determined by ultrasound: a study in Gezira, Sudan. Am J Trop Med Hyg 39: 196–201.
- Vennervald BJ, Kenty L, Butterworth AE, Kariuki CH, Kadzo H, et al. (2004) Detailed clinical and ultrasound examination of children and adolescents in a *Schistosoma mansoni* endemic area in Kenya: hepatosplenic disease in the absence of portal fibrosis. Trop Med Int Health 9: 461–470.
- Wilson S, Vennervald BJ, Kadzo H, Ireri E, Amaganga C, et al. (2007) Hepatosplenomegaly in Kenyan schoolchildren: exacerbation by concurrent chronic exposure to malaria and *Schistosoma mansoni* infection. Trop Med Int Health 12: 1442–1449.
